# Opposing Effects of *Ceanothus velutinus* Phytochemistry on Herbivore Communities at Multiple Scales

**DOI:** 10.3390/metabo11060361

**Published:** 2021-06-07

**Authors:** Casey S. Philbin, Matthew Paulsen, Lora A. Richards

**Affiliations:** 1 Hitchcock Center for Chemical Ecology, University of Nevada, Reno, NV 89557, USA; mattpaulsen950@gmail.com (M.P.); lorar@unr.edu (L.A.R.); 2 Department of Biology, University of Nevada, Reno, NV 89557, USA

**Keywords:** metabolomics, community ecology, chemical ecology, mass spectrometry, *Ceanothusvelutinus*, lepidoptera, elevational gradient

## Abstract

Identifying the interactions of functional, biotic, and abiotic factors that define plant–insect communities has long been a goal of community ecologists. Metabolomics approaches facilitate a broader understanding of how phytochemistry mediates the functional interactions among ecological factors. *Ceanothus velutinus* communities are a relatively unstudied system for investigating chemically mediated interactions. *Ceanothus* are nitrogen-fixing, fire-adapted plants that establish early post-fire, and produce antimicrobial cyclic peptides, linear peptides, and flavonoids. This study takes a metabolomic approach to understanding how the diversity and variation of *C. velutinus* phytochemistry influences associated herbivore and parasitoid communities at multiple spatiotemporal scales. Herbivores and foliar samples were collected over three collection times at two sites on the east slope of the Sierra Nevada Mountain range. Foliar tissue was subjected to LC-MS metabolomic analysis, and several novel statistical analyses were applied to summarize, quantify, and annotate variation in the *C. velutinus* metabolome. We found that phytochemistry played an important role in plant–insect community structure across an elevational gradient. Flavonoids were found to mediate biotic and abiotic influences on herbivores and associated parasitoids, while foliar oligopeptides played a significant positive role in herbivore abundance, even more than abundance of host plants and leaf abundance. The importance of nutritional and defense chemistry in mediating ecological interactions in *C. velutinus* plant–herbivore communities was established, justifying larger scale studies of this plant system that incorporate other mediators of phytochemistry such as genetic and metageomic contributions.

## 1. Introduction

The phytochemical phenotype of an individual plant is the culmination of an evolutionary history within the context of trophic and abiotic interactions throughout its life history. As these interactions vary across the landscape, it is predicted that phytochemistry will similarly vary spatially and temporally [1]. Understanding the scale at which plant chemistry varies and how this variation mediates interactions is an emerging focus within community chemical ecology. Phytochemistry can be quantified across multiple dimensions from single compounds and subsets of compounds to the diversity of the full chemical profile. These chemical dimensions can vary at hierarchical spatial scales across the landscape, such as between individuals within plots, at the plot level, and at the population level [2]. Phytochemistry is also known to vary temporally at multiple scales in response to multiple factors [3]. As plants interact with their surroundings through chemistry, illuminating the chemical linchpins underlying the plant community structure reveals the mechanisms through which plants orchestrate ecosystem dynamics.

Plants can have phytochemical responses to bottom-up stimuli, such as abiotic factors and the epiphytic microbiome, or top-down stimuli such as herbivory. Since the early adaptations to terrestrial life, plants have evolved suites of specialized compounds to deal with abiotic stress. The diversity of structures and functions of flavonoids seen today come from ancient biosynthetic pathways that evolved as UV protectants [4,5]. While flavonoids now have diverse functional roles in plants, including antifungal, allelopathy, pigmentation, and antiherbivore functions [6,7,8], they are one of the most ubiquitous compound classes and serve primarily as protectants to abiotic stress [9,10]. For example, flavonoid concentrations have been found to increase seasonally [11,12,13] and with elevation [11,12,13]. These spatial and temporal changes are thought to be in response to variation in abiotic conditions. Phenotypic variation between and within species contribute to the diversity of interactions between plants, herbivores, and, subsequently, parasitoids [14]. For example, interspecific phytochemical variation has been found to predict herbivore assemblages [15,16]. At a finer taxonomic scale, the chemical variation within a species can effectively predict herbivore presence [17] and community assemblage [18]. At higher trophic levels, intraspecific variation in phytochemistry can influence parasitoid performance [19] and attack [20]. Phytochemical variation is key to mediating indirect effects of abiotic factors on plant–insect communities and is essential to understanding ecosystem dynamics.

The onset of metabolomic approaches to community-level chemical ecology has allowed research to leverage natural phytochemical variation to statistically narrow in on meaningful patterns [21]. While ecological studies tend to have many more subjects (10^2^–10^3^) than traditional metabolomics experiments (<100) [22], there remains a number of subjects versus the number of variables problem that requires dimension reduction and summarization for many statistical approaches. Advances have been made in developing experimentally meaningful composite variables for studying ecological interactions, including ordination, networking, and classification techniques [17,18,23,24,25]. Another useful approach is the quantitation of chemical diversity, a measure of the richness (number of compounds) and evenness (compound abundance) of phytochemical mixtures drawn from community ecology [26,27,28]. The phytochemcial diversity of a mixture results from two intermingled dimensions of diversity: compositional diversity (the richness and evenness of individual compounds) and structural complexity (the size and topological complexity of constituent molecules in a mixture, as measured by ^1^H NMR). For some phytochemical systems, measuring the variation in structural complexity may be more relevant than compositional diversity [28]. Another important chemical diversity concept drawn from community ecology is beta diversity, or species turnover [5,29]. From a chemical standpoint, this represents the variation of phytochemical composition across spatial or temporal scales. Identifying the scale at which phytochemical diversity and composition has the strongest variance can indicate which ecological processes are being mediated through chemistry.

The application of such composite variables is useful for summarizing chemical variation but untangling and interpreting the contributions of individual compounds to these variables can be complicated. Methods have recently been developed in our lab for applying correlation networks to metabolomics data using weighted gene co-expression network analysis (WGCNA) [30], an approach which generates co-expression networks of covarying phytochemicals from which modules, highly interconnected discrete clusters of covarying chemical signals, are generated [23,24]. The first principal component of the compounds within a given module yields an eigenvalue for each module, which is a composite variable that can be applied to statistical analyses. The chemical signals that contribute most to a given module can be readily determined from the loadings on the eigenvalue, or by determining the most highly connected compounds within a module [30]. By partitioning the metabolome based on covariance network modules before principal components analysis, this approach yields composite variables that represent linear combinations of unambiguous groups of compounds whose contributions are much easier to interpret. Although we did not annotate individual compounds beyond phytochemical class in this study, this network-based approach allows us to not only determine the importance of chemistry within plant–insect communities, but to readily determine which *individual compounds* are involved in ecological interactions.

**Figure 1:**
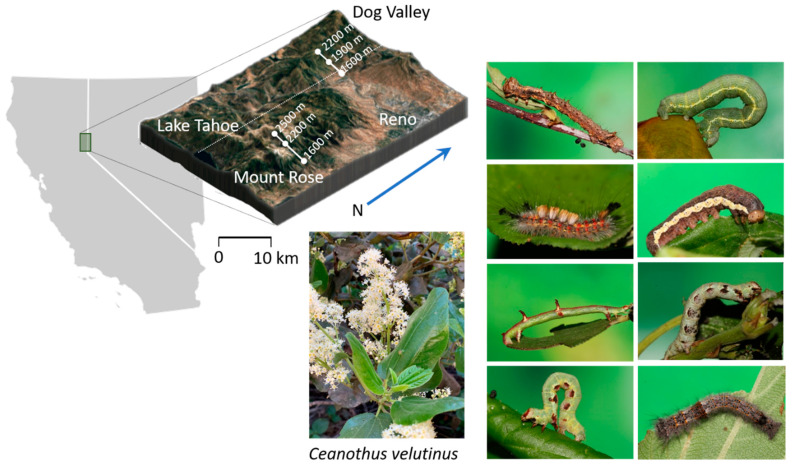
Collection site locations in the eastern Sierra Nevada, host species, and representative herbivores. 3D surface map includes 10 km scale for reference.

This study focuses on *Ceanothus velutinus*, an evergreen shrub found across western North America, growing readily in multiple habitats from high desert to coniferous forests (Figure 1). *Ceanothus velutinus* is a fire-adapted species and much of the research on this species is focused on its role post-fire [31,32], its contributions to soil nitrogen availability [33,34,35,36], its symbiotic relationships with *Frankia* [37], and its leaf trait evolution [38]. Given the abundance, distribution, and its role establishing nitrogen availability post-fire, *C. velutinus* will likely have an influential role in the plant–insect communities that eventually develop around it. Despite this, there are surprisingly few studies investigating herbivore interactions [32,39,40]. On the west slope of the Sierra Nevada Mountains, the predominant herbivores found are *Agronus cinerarius*, *Drepanulatrix foeminaria*, and *Nymphalis californica* [39,41], but *N. californica* are rare on the east slope of the Sierra Nevada [41], where *C. velutinus*-associated herbivores remain largely unstudied. With the increasing frequency of fire incidents on public forest land in the American west, understanding how plant–insect communities develop in the aftermath of these incidents will be critical for conservation and management efforts. Discovering phytochemical predictors of community parameters can provide early indicators of habitat quality after ecological perturbations [42]. While some previous work has found *Ceanothus* to produce flavonoids [43,44] and cyclopeptide alkaloids [45,46,47], there have been no studies to investigate *Ceanothus* phytochemistry in biotic and abiotic interactions. In addition to recording the natural history of *C. velutinus* communities on the eastern slope, this study intends to determine phytochemical biomarkers that influence the abundance and diversity of lepidopteran herbivores and their associated parasitoids by applying a hierarchical approach to the community chemical ecology outlined above: (1) determine the ecologically relevant spatiotemporal scale and dimensions of chemical diversity; (2) generate composite variables to summarize phytochemistry diversity and variation; (3) apply the composite variables to statistical models predicting ecological interactions; (4) annotate the chemical composition of ecologically relevant composite variables to the level of phytochemical class. This investigation will reveal the biotic and abiotic factors that underly variation in *C. velutinus* chemistry, and ultimately affect higher trophic levels.

## 2. Results

### 2.1. Modeling Tritrophic Interactions in C. velutinus Communities of Dog Valley and Mt. Rose

**Figure 2:**
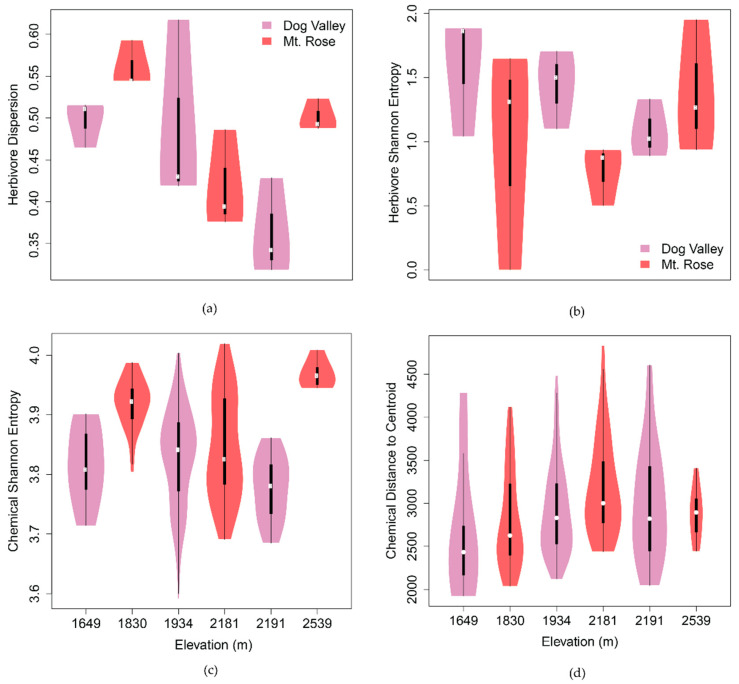
Subsite-level dispersion and diversity across elevation of herbivores and phytochemistry of *C. velutinus* communities. (**a**) Inter-plot herbivore dispersion (ANOVA_5,12_; *p* < 0.05) differed significantly among subsites, while (**b**) mean plot herbivore diversity (Shannon entropy; ANOVA_5,12_; *p* = 0.37) did not. (**c**) Phytochemical diversity (ANOVA_5,295_; *p* < 0.001) and (**d**) dispersion (ANOVA_5,295_; *p* < 0.05), aggregated by month, individual, and plot, varied significantly among subsites. Herbivore dispersion and phytochemical diversity have a sinusoidal relationship with elevation, having a crest at ca. 1830 m and trough at ca. 2191 m. Across elevation, herbivore diversity and chemical dispersion have an inverse mid-domain effect relative to one another, with herbivore diversity having a valley and chemical diversity having a peak at ca. 2200 m a.s.l.

We investigated two populations of *C. velutinus* and their associated herbivores and parasitoids along the eastern slope of the Sierra Nevada, Dog Valley, and Mt. Rose (Figure 1). These populations differed in their plant age, herbivores, and phytochemical structure. The two sites have similar total estimated leaf abundance (Dog Valley: 1.03 × 10^5^; Mt. Rose: 9.98 × 10^4^), however, Dog Valley plots had a higher number and variance of plants per plot (25 ± 19 [SE]) with less leaves per plant (1377 ± 332 [SE]) compared to Mt. Rose with less plants per plot (10 ± 4 [SE]) and a higher number and variance of leaves per plant (3325 ± 1479 [SE]), including the plant with the highest estimated number of leaves (4 × 10^4^). As the number of leaves on a plant are relative to plant age, Dog Valley has a younger *C. velutinus* population, and especially at higher elevations, Mt. Rose has an older population. The *C. velutinus* composition of study plots is summarized in Table S1. There appeared to be a mid-domain effect [48] on the mean number of trees per subsite and an inverse mid-domain effect observed in the number of leaves per subplot (Figure S1). The lepidopteran herbivore community of these plants was surveyed three times from May to late August in 2019 using a standardized beat sheet method and resulted in a total 542 lepidoptera, with 408 from Dog Valley and 134 from Mt. Rose. The herbivores associated with *C. velutinus* at these sites include 19 distinct morphotypes. The morphotypes were based on both larval and adult morphology. Many were identified to the subfamily level or higher (*Eupithecia nevadata, Malacosoma californica, Nola minna, Satyrium saepium, Hesperumia fumosaria, Orgyia vetusta, Phyllodesma Americana, Egira perlubens Nymphalis californica, Drepanulatrix monicaria*). Some caterpillars could not be morphotyped due to dying during rearing or being collected dead but are included here in total herbivore abundance. Although herbivore dispersion (spatial variation in herbivore community composition) was equivalent between sites (ANOVA_1,16_; *p* = 0.76) composition differed significantly (ADONIS_1,16_: *p* < 0.01, Figure S4). Herbivore dispersion varied significantly at the subsite level across elevation (Figure 2, ANOVA_5,12_; *p* < 0.05), but diversity did not (Figure 2, ANOVA_5,12_; *p* = 0.37). The larvae were collected and reared resulting in an adult lepidopteran, or if the larvae was parasitized, the emergence of an adult parasitoid. The frequency of parasitoid success was calculated as the percentage of parasitoid-yielding lepidoptera from the total of larvae that survived to adult or parasitoid emergence. The frequency of parasitoid success at Mt. Rose (59%) was more than three-fold higher for Dog Valley (19%).

**Figure 3:**
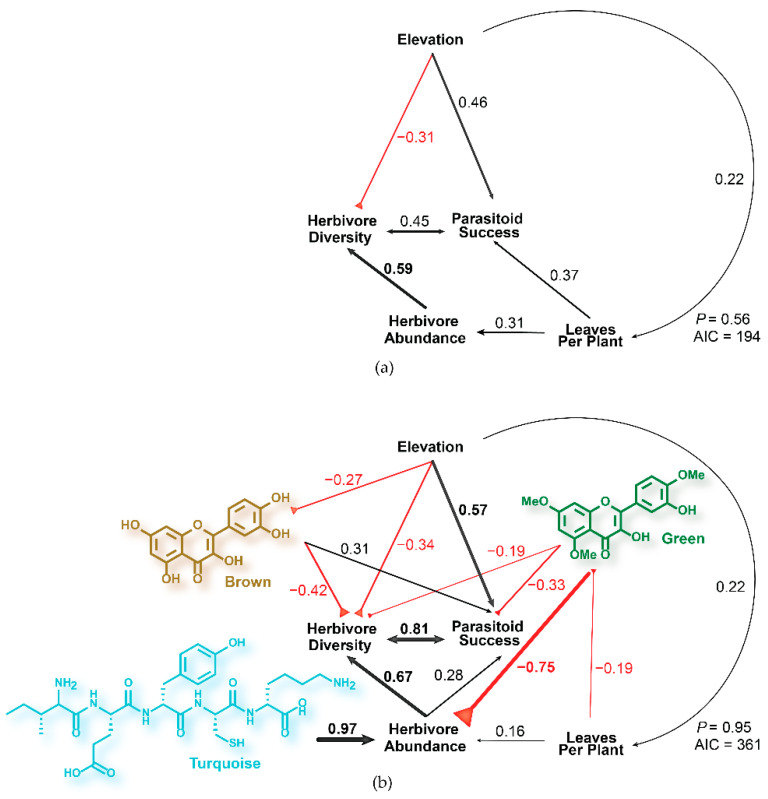
(**a**) Structural equation model predicting the effect of elevation on parasitoid success, herbivore diversity, and abundance mediated by *C. velutinus* leaves per plant within plots. (**b**) Structural equation model predicting herbivore diversity and abundance based on plant properties, abiotic factors, and chemical modules. The SEM incorporating chemical variables explains 33% more variance than the model without chemistry. Brown and green modules represent flavonoids, known defensive compounds, while the turquoise module represents peptides, a possible indicator of nutritional quality and nitrogen content.

To understand how elevation predicts community parameters at the plot level, a structural equation model was constructed (Figure 3a). Total number of plants, total leaf abundance, and leaves per plant were all tested as measures of *C. velutinus* abundance, and leaves per plant gave the best model fit. There was a 56% probability that this model agrees with observed data (based on the Χ^2^ goodness of fit test [49]), with the strongest relationship between herbivore abundance and herbivore diversity (coeff. = 0.59) indicating that plots with high herbivore abundance also had high herbivore diversity. Surprisingly, parasitoid success had a strong positive covariance (coeff. = 0.45) with herbivore diversity and not herbivore abundance. Plots with larger plants hosted more diverse and abundant herbivore communities (coeff. = 0.27 and 0.31, respectively), as well as higher numbers of successful parasitoids (coeff = 0.37). *Ceanothus velutinus* leaves per plant (coeff. = 0.22) and parasitoid success (coeff. = 0.46) were directly increased by elevation, while herbivore abundance was indirectly increased through leaves per plant. Elevation had a direct negative effect on herbivore diversity (coeff. = −0.31), despite having positive indirect effects through all other biotic factors. 

### 2.2. Chemically Mediated Ecological Interactions in C. velutinus Communities

To examine if and how phytochemistry mediates multitrophic interactions in *C. velutinus* communities, the relevant composite variables to summarize chemical variation across multiple scales were determined. 

#### 2.2.1. Spatiotemporal Chemical Variation

To capture temporal chemical variation, leaf samples were collected three times through the growing season. Chemical diversity was calculated as the Shannon effective number of peak bins for individual plants within each collection month, which ranged from 31.7 to 57.6 effective peaks. Mean chemical diversity was higher (ANOVA_1,299_; *p* < 0.001; Figure S4) at the Mt. Rose site (49 ± 1 [SE] effective peaks) than at the Dog Valley site (45 ± 1 [SE] effective peaks). Mean chemical diversity also varied significantly among subsites (ANOVA_5,295_; *p* < 0.001; Figure 2), plots (ANOVA_104,196_; *p* < 0.001), months within-plot (ANOVA_53,247_; *p* < 0.001), and individuals averaged across months (ANOVA_5,295_; *p* < 0.001). Mean chemical diversity aggregated at all levels showed a sinusoidal relationship to elevation (Figure 2, Figure S5).

The distance-based methods of a betadisper and ADONIS in the vegan R package [50] were used to compare chemical dispersion (spatiotemporal variance of the chemical profile) and composition (the chemical make-up of individual plants) across scales within and between sites. While chemical dispersion and composition did not vary significantly between sites (ANOVA_1,299_; *p >* 0.05, Figure S4), chemical dispersion differed between subsites (ANOVA_5,295_ *p* < 0.05), and those differences appeared to have an elevational structure. The peak in chemical dispersion near 2 km elevation, with a concomitant valley in herbivore dispersion (Figure 2), suggests there may be a mid-domain effect on chemical diversity and herbivore diversity over this elevational gradient [48]. Inter-monthly chemical dispersion within individuals (ANOVA_104,196_ *p* < 0.001, Figure S6) and inter-individual chemical dispersion between collection times (ANOVA_17,283_ *p* < 0.05) were both significant (Figure S6) within subsites (as some plots consisted of a single plant, this could not be calculated at the plot level), which suggests that plot-level variation in chemistry is driven by both temporal and interindividual differences in chemistry (Figure S5). 

At the subsite level, herbivore dispersion was negatively (*r* = −0.60, *p* < 0.05) correlated with elevation and positively correlated (*r* = 0.73, *p* < 0.01) with phytochemical diversity, while herbivore diversity was positively correlated (*r* = 0.69, *p* = 0.10) with natural log-transformed leaf abundance and negatively correlated (*r* = −0.66, *p* = 0.21) with phytochemical dispersion, although these were not significant correlations. No correlation was observed between herbivore abundance and herbivore diversity (*r* = 0.12), herbivore dispersion (*r* = 0.33), or elevation (*r* = 0.20) at the sublocation level. A linear model was constructed (R^2^ = 0.94, *p* < 0.01) wherein herbivore dispersion was predicted by phytochemical diversity (m = 1.0, *p* < 0.01) and elevation (m = −1.6 × 10^−04^, *p* < 0.05). 

#### 2.2.2. Phytochemical Covariance Clusters

**Figure 4:**
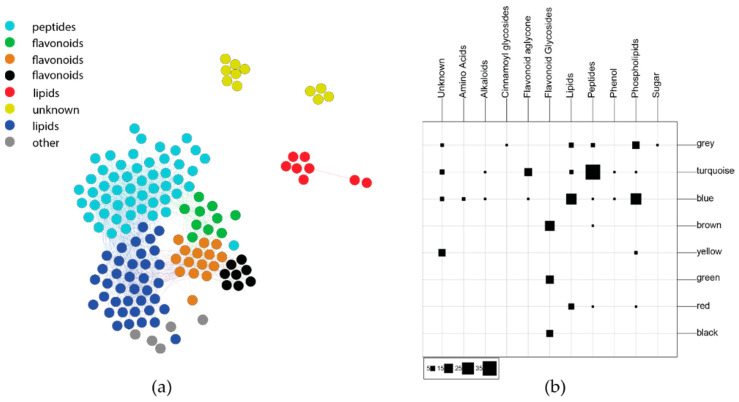
(**a**) WGCNA network of covarying *C. velutinus* compounds. The brown and green modules represent flavonoid glycosides, while the turquoise module largely represents oligopeptides. (**b**) Confusion matrix comparing WGCNA modules and phytochemical class.

Diversity- and distance-based composite variables are useful for exploring how chemical variation holistically interacts with ecological factors, but untangling how individual compounds contribute to these variables can be difficult. To determine which phytochemicals were important to ecological interactions, composite variables were generated that grouped compounds across the chemical profile that could be used in subsequent statistical analyses. A correlation network-based approach (WGCNA) [24,30] was applied to reduce the dimensionality of our chemistry data into clusters (network modules) of cooccurring and covarying compounds. Eigenvalues summarize the variance of each group of compounds (modules) and the relative weight of each module on individual samples. The analysis yielded eight modules, each representing a different group of covarying compounds (Figure 4, left). Phytochemical peak bins were partitioned by module color to determine which module had the highest influence on plot-level dispersion (Figure S8). The black module did not have significant between-plot dispersion (ANOVA_17,431_; *p* > 0.1), but all other modules showed significant dispersion (ANOVA_17,431_; *p* < 0.01). The highest F-statistic values were found for the brown, turquoise, and green modules (ANOVA_17,431_; *F*_brown_ = 13.9, *F*_green_ = 4.7, *F*_turquoise_ = 4.3), while the remaining modules had *F*-statistic values ranging from 2.3 to 3.5.

The annotations described above were used to determine the predominant metabolite classes represented by the WGCNA modules. The confusion matrix in Figure 4 shows which compound classes are represented within each module. The modules largely agree with compound class, which is not always the case, as multiple compound classes may be co-regulated as a result of biotic and abiotic stress [17]. The turquoise module is predominantly comprised of peptides, along with several flavonoid aglycones. The blue, red, and yellow modules represent the most lipophilic compounds including lipids and phospholipids, as well as some late-eluting compounds that could not be annotated. The brown, green, and black modules are almost totally comprised of flavonoid glycosides. Based on database matches, the most highly represented flavonoid matches in the brown and green modules were polyhydroxylated flavanone glycosides, with methoxylated flavanones found more often in the green module. 

### 2.3. Phytochemical Models of Tritrophic Interactions in C. velutinus Communities

To determine how chemistry mediates multitrophic interactions, modules were incorporated into the structural equation model in Figure 3a. For the initial model, all chemical variables were added as contributing to all herbivore and parasitoid variables, and the elevation and plant age were added as contributing to all chemical variables (*p* = 0.51, AIC = 374). The least significant chemical variables were iteratively removed, and non-significant elevation and plant age variables were removed as contributors to chemical variables until the best fitting model was reached. The gray module was not included in any models due to its low variance. Although the new model is more complex than the simpler model (Figure 3a), due to the incorporation of additional variables and interactions (AIC = 361 vs. 194 for the simpler model), the model had a higher probability (95%) of agreeing with the observed data in *C. velutinus* communities (*p* = 0.95, Figure 3a). The peptide module had a strong positive direct effect on herbivore abundance across plots (coeff. = 0.97), and the direct effect of leaf abundance (coeff. = 0.16) was diminished over the previous model. In contrast, the green flavonoid module had a large negative direct effect on herbivore abundance (coeff. = −0.75), despite the strong positive correlation with peptides (coeff. = 0.85). This module also had direct negative effects on herbivore diversity (coeff. = −0.19), with indirect effects mediated through herbivore abundance, and parasitoid success (coeff = −0.33), with indirect effects mediated through both herbivore abundance and diversity. Leaves per plant (a proxy for plant size and age) had direct negative effects on the green flavonoid module (coeff. = −0.19), which mediated indirect positive effects on herbivore abundance. Elevation had direct negative effects on the brown flavonoid module (coeff. = −0.27), which mediated the indirect effects of elevation on herbivore diversity. The brown flavonoid module, which correlates with both the green (coeff = 0.49) and turquoise modules (coeff. = 0.46), had a stronger direct negative effect on herbivore diversity (coeff. = −0.42) than the green flavonoid module but had direct positive effects on parasitoid success (coeff. = 0.31). The direct effects of elevation on leaves per plant (coeff. = 0.22) and herbivore diversity (coeff. = −0.34) remain largely unchanged from the previous model, but after incorporating chemistry the direct effect of elevation on parasitoid success increased (coeff. = 0.57).

## 3. Discussion

This study has established the importance of phytochemical diversity and variation in the context of biotic and abiotic factors in determining the composition of *C. velutinus* herbivore and parasitoid communities. Our initial model excluding chemistry (Figure 3a) showed that plots with larger, more established plants had higher levels of herbivore diversity, abundance, and parasitoid success, largely driven by increases in elevation. Seed dispersion coming from the west slope of the Sierra Nevada might lead to more mature *C. velutinus* populations establishing earlier at higher elevations. However, elevation directly reduced herbivore diversity, despite the positive indirect effects, mediated through plant size and parasitoid success. Pellissier et al. [51] found that lepidoptera populations decrease in diversity and abundance with increasing elevation in alpine ecosystems, resulting in an increased diet breadth for the higher elevation lepidopterans. This was consistent with our results that herbivore turnover steadily decreases with elevation (Figure 2) with the exception of the highest and lowest sublocations, which also have the highest leaf abundance (Figure S1b). In this context, then, the increases in leaf abundance (and the resulting increase in herbivore abundance) across elevation outweigh the effects of elevation directly suppressing herbivore diversity through stresses encountered at high altitude [52].

The nonlinear effects of elevation on herbivore diversity and phytochemical dispersion indicated a mid-domain effect [48] for both. Herbivore diversity was the lowest at approximately 2200 m a.s.l. elevation, with a concomitant increase in phytochemical dispersion (Figure 2). Mid-domain effects on plant community diversity have been observed over elevational gradients, similar to this study, in alpine systems [52] and for moth species richness in a tropical rain forest [53]. This minimum in herbivore diversity may also be due to low total leaf abundance at mid-elevation (Figure S1). Mid-elevation plots with fewer leaves may be unable to host more diverse herbivore populations. Chemical dispersion at subsite scale captures interindividual, inter-plot and temporal variation in chemistry. With higher numbers of plants at this elevation, peak mid-elevation chemical dispersion may result from higher interindividual differences in chemistry (Figure S1). The lower number of leaves in these plants also indicates younger plants that might have more variable chemistry in response to temporal variance in temperature, rainfall, sunlight, and other abiotic factors known to change along elevational gradients [52]. Dynamic chemistry in these mid-elevation plants may then also deter specialist herbivores, and be more amenable for generalists, reducing herbivore diversity across time, similar to the effect of metabolic complexity on specialists and generalists associated with *Piper* [26]. This relationship supports the screening hypothesis that phytochemical variation is maintained to increase the probability of producing effective combinations of defensive compounds [54].

In contrast to the mid-domain distribution of herbivore diversity, herbivore dispersion had a sinusoidal relationship to elevation that was matched by phytochemical diversity. Herbivore dispersion decreased with elevation, but this relationship may have been convoluted by the high abundance and variance of leaves at the highest elevation subsite (Figure S1). A similar pattern was observed for chemical diversity that may also be explained by the makeup of the plant community at the highest elevation subsite. The variance in leaf abundance suggests a mixture of established and younger plants at this site that may contribute to more diverse chemistry at this subsite. While decreases in chemical defenses have been observed with increasing elevation [51], this was attributed to a decrease in herbivore abundance with elevation, and this study observed no relationship between elevation and herbivore abundance. This suggests that variation in herbivore communities is driven by elevation, mediated through chemical diversity. These non-linear relationships with elevation may also result from differences in the plant composition and other environmental variables between the two sites, leading to the herbivores and the phytochemistry having different elevational relationships at the two sites. We will examine this further in subsequent studies with higher numbers of elevational transects across a variety of communities having similar and dissimilar environs.

Changes in phytochemical variation in determining herbivore community composition became clear when chemistry was incorporated into the model as modules representing covarying groups of phytochemicals (Figure 3b). Peptides had the strongest influence on herbivore abundance, suggesting that peptides may provide a nutritional component of the herbivore diet. *Ceanothus* spp. are known to produce cyclic peptides found in their root nodules [45,46,47]. Linear precursors of these cyclic peptides have been found in cell cultures of *Ceanothus americanus* L. [55], suggesting that precursors are produced in the foliar tissues before being converted to macrocyclic peptides in root nodules which host diverse actinorhizal endophyte communities [56]. Herbivores may then be reaping the nutritional rewards of the endophytic processes driving peptide production in the rhizosphere [57,58], regardless of other environmental factors. Leaves higher in nitrogen are known to increase larval growth rates and fecundity [59,60], and depending on their amino acid content, these short chain peptides may prove to be a high-quality source of nitrogen for herbivores [61]. Given the importance of these peptides to herbivore abundance in *C. velutinus*, future studies should include measures of nodulation and endophyte diversity.

The effects of diet quality derived from oligopeptides are offset by covarying defensive flavonoids represented by the brown and green modules (Figure 3b). Although the green module flavonoids covary with peptides, they had a strong negative direct effect on herbivore abundance and negative effects on herbivore diversity and parasitoid success. Flavonoids are known to be important to plant resistance [8], and a broad array of flavonoids have been found across multiple species of *Ceanothus* [44]. The negative influence of plant size on the green module flavonoids suggests that less developed plants may be more susceptible to more diverse herbivore communities at lower elevations, requiring improved defenses [51]. While both the green and brown flavonoids directly suppress herbivore diversity, the brown module flavonoids directly increase parasitoid success. This could possibly indicate that these flavonoids are acting as immunosuppressants for these herbivores, which leads to increased parasitoid success [62]. Alternatively, parasite infected herbivores could be improving their survival by consuming plants higher in antioxidants [63], a long-established function of flavonoids [64]. We do not know the extent to which the dead caterpillars in our study were parasatized, so we cannot make this conclusion. Elevation had direct negative effects on brown module flavonoids, which is unusual as flavonoids are generally found to increase with elevation due to their role as UV protectants [6,8,13]. However, the concentrations required for plant defense in response to higher herbivore diversity at lower elevations may supersede that required for UV protection at these elevations. Additionally, there may be other abiotic factors driving flavonoid accumulation in this system, such as heat, temperature, and osmotic stress that may also differ between the two sites. As plant development may also influence flavonoid accumulation, the differences in plant age between sites may also factor into flavonoid response to abiotic factors, although this was accounted for in path models by leaves per plant, which was not found to affect green flavonoid levels.

Here we have demonstrated how phytochemistry mediates, and sometimes acts independently of, ecological factors in shaping herbivore communities of *C. velutinus* and their associated parasitoid populations. We established which spatiotemporal relationships were important to uncovering meaningful variation in herbivores and phytochemistry. This study was conducted outside the host range of *Nymphalis californica* (California Tortoiseshell) [41], one of the predominant lepidopteran predators of *C. velutinus* [39], which can cause heavy defoliation during outbreaks. However, anthropogenic climate change may force *N. californica* into new host ranges as has been observed for *Danaus plexippus* [65]. By establishing baseline parameters for the herbivore populations and the phytochemistry of *C. velutinus* communities pre-range expansion, we can better understand which chemical, biotic, and abiotic factors contribute to site selection.

## 4. Materials and Methods

### 4.1. Study Site 

*Ceanothus* (Rhamnaceae) is a species-rich North American genus of woody perennial that includes about 55 species, with 51 species found in Western North America [66] and with the highest diversity (38 species) and endemism in the California Floristic Province [67,68]. *Ceanothus* have symbiotic relationships with nitrogen-fixing bacteria [57] and play an important role in ecosystem nitrogen availability [66]. *Ceanothus velutinus* (Tobacco brush) was chosen as the focal species for this study. *C. velutinus* is typically found at elevations between 1600 m a.s.l. and 2500 m a.s.l. and at times in very dense stands. Eighteen plots were established across two sites on the Eastern slope of the Sierra Nevada, referred to as the Dog Valley (39°31′39.12″ N, 120° 1′53.40″ W) and Mt. Rose locations (39°20′33.55″ N, 119°52′11.33″ W). Both sites are characterized by eastern facing slopes with Dog Valley having a lower minimum elevation (1632 m a.s.l.) and slope (46 m/m) and Mt. Rose having a higher maximum elevation (2550 m a.s.l.) and slope (145 m/m). The plots were 10 m in diameter and set up in triplicate along an elevational gradient between 1600 m a.s.l. and 2600 m a.s.l. at “low”, “medium”, and “high” elevation bands (referred to here as subsite). Elevation for each band was established relative to the elevational range of each site, ±50 m. Each plot was at least 10 m away from any trail or road, spaced at least 100 m apart from any other plot, and contained at least one *C. velutinus* individual, chosen as the center point of the plot. The plots were established in 2018. Ecological and chemical data were collected on the same day from each plot monthly from May to October of 2018. Due to the extra time involved with locating sites and setting up the plots, we repeated data collections in 2019 to ensure we captured a complete dataset for all sites over the season. 

### 4.2. Ecological Data

For each plot, the total plant species diversity and the estimated leaf abundance of each plant within the plot was determined. Leaf abundance was estimated by counting the leaves on two representative branches from a given plant, then estimating the number of such branches on the plant. Each *C. velutinus* individual within the plot was numbered and the lepidoptera were collected using a beat sheet. Voucher samples were collected for all *C. velutinus* individuals and are available from the herbarium at the University of Nevada, Reno Natural History Museum. All collected caterpillars were reared individually in plastic cups in the lab at UNR to either adult or parasitoid emergence. Host plant foliage was replaced every 2 days and all pupae were checked daily. The adult Lepidoptera and parasitoids that emerged from the pupae were allowed to fully harden and then placed in a freezer for storage before pinning and identification. Each caterpillar collected was photographed and assigned a unique voucher code, linking the individual to the plot and plant number in the database. Morphotypes were later condensed and validated using reared adult specimens and larval photographs as references. 

### 4.3. Sample Preparation

Immediately after caterpillars were collected using a beat sheet, leaf samples were collected from each *C. velutinus* individual for phytochemical profiling. Young leaves were cut from the terminal end of the branches, placed in paper bags, and stored in a cooler with dry ice until the end of the collection day, and subsequently stored at −80 °C. Leaves were then transferred to plastic centrifuge tubes with a tungsten steel bead, lyophilized, and ground to a fine powder at 30 Hz for two minutes using a tissue lyser (Qiagen Tissuelyser II; Hilden, Germany). The ground plant material (~20 mg) was transferred to screw-cap scintillation vials with 1 mL of 70% aq solution of HPLC-grade, denatured ethanol (Fisher, Pittsburgh, PA, USA) in 18 MΩ water. The samples were vortexed, sonicated for 10 min and incubated overnight on a shaker at room temperature and randomized before filtration through a 96 well filter plate with 1.0 mL capacity and 1.0 μm glass fiber filters (Acroprep 96, Pall Corporation, Port Washington, NY, USA) into a 96 well plate with 1 mL glass inserts, sealed with a silicone cap mat, and stored at −20 °C for less than one week until analysis. 

### 4.4. LC-TOF Analysis of Foliar Plant Tissue

Chromatography was performed on an Agilent 1200 analytical HPLC equipped with a binary pump, autosampler, column compartment and diode array UV detector, coupled to an Agilent 6230 Time-of-Flight mass spectrometer via an electrospray ionization source (ESI-TOF; gas temperature: 350 °C, flow: 8 L/m; nebulizer pressure: 35 psig; VCap: 3500 V; fragmentor: 175 V; skimmer: 65 V; octopole: 750 V). Internal standards were selected to represent the major metabolite classes found in *Ceanothus*: flavonoid glycosides (narignin), flavonoid aglycones (naringenin), and peptides (cholecystokinin fragment), but were not found in plants. Multiple internal standards were used to account for differences in ionization efficiency among phytochemical classes. Extracts (1.00 μL) were co-injected with 1.00 μL of internal standard mix (naringenin, 100 μM; naringin, 101 μM, Cholecystokinin Fragment 30–33 Amide, (52 μM) Sigma-Aldrich) and eluted at 0.400 mL/min through a Kinetex EVO C18 column (Phenomenex, 2.1 mm × 100 mm, 2.6 μ, 100 Å) at 40 °C. The linear binary gradient was comprised of buffers A (water containing 10 mM ammonium acetate) and B (acetonitrile) changing over 25 min accordingly: 0 min 5% B, ramp to 40% B at 8 min, ramp to 100% B at 14 min, 14–20 min 100% B ramping to 0.70 mL/min before re-equilibrating the column from 21–25 min at 5% B, 0.4 mL/min.

The Agilent-formatted raw data were converted to mzML format in ProteoWizard MSConvert 3.0 [69] before processing using the Bioconductor package XCMS [70] in R. Peaks were extracted using the centwave function before retention time correction using obiwarp, peak density correspondence analysis, and gap-filling to yield 1308 aligned retention time and *m*/*z* bins (chromatographic features). Features were visually inspected to remove 260 peaks representing areas of baseline noise above peak threshold. CAMERA [71], an XCMS wrapper, was then used to identify groups of features (pseudospectra) having similar retention time and were correlated across samples (r > 0.7), with similar (r > 0.5) peak shape and by isotopic pattern. The CAMERA command *annotate* was used to identify and remove features resulting from ammonium adducts that might lead to the misidentification of nitrogenous compounds. The feature within a pseudospectrum group with the highest abundance was then chosen to represent the peak area of each of the 300 resulting pseudospectra. To minimize between-run batch effects, each peak bin was subjected to ANOVA analysis grouped by batch and 133 peaks exhibiting significant batch effects (*p* < 0.10) were excised. 

### 4.5. Compound Classification, Annotation, and Normalization

Gaussian model clustering was executed using the mclust package [72] in R to generate clusters of compounds sharing similar chromatographic and mass spectrometric properties that represent presumptive compound classes. Before cluster analysis, the 177 peaks remaining after quality control filtering were partitioned into even- and odd-mass ions to separate presumed nitrogenous compounds from other compounds based on the nitrogen rule. It was assumed that some odd-mass ions did not completely exclude nitrogenous compounds if they had even numbers of nitrogen. Cluster models and number of clusters were selected according to the Bayesian information criterion for nitrogenous and non-nitrogenous compounds based on retention time (rt), charge-to-mass ratio (*m*/*z*), and relative mass defect (RMD) for each compound. Relative mass defect is calculated based on the difference between the high-resolution mass and nominal mass of a given compound. Higher RMD indicates a higher proportion of hydrogen atoms in the chemical formula of that compound [23].

Phytochemical peak bins were annotated to identify those to be normalized to the internal standards. Peak bins were partitioned into odd *m/z* and even *m/z* peaks, which were presumed to be nitrogenous compounds based on the nitrogen rule. The odd and even *m/z* sets of peak bins were subjected to separate Gaussian cluster analyses, which resolved nine and seven clusters of compounds respectively, based on their rt, *m/z*, and RMD (Figure S2). The peak bins were classified as peptides (40 peaks), flavonoid glycosides (35 peaks), phospholipids (33 peaks), lipids (31 peaks), phospholipids (33 peaks), flavonoid aglycones (11 peaks), several minor classes (7 peaks), and several peaks that could not be identified (18 peaks). While some clusters represented mixtures of chemical classes, internal standards were grouped into clusters with the same predominant chemical class (Figure S3). The mean rt, *m/z*, and RMD of these clusters, and their predominant putative compound class are reported in the supporting information (Table S2). Even-mass cluster six was removed because all peak bins contained therein were excised during quality control filtering. The putative peptides were characterized as having a high variance of rt, *m/z*, and RMD, which presumably depends on their primary structure and amino acid composition. The smaller (low *m/z*) peptides tended to have lower retention times and RMD. The flavonoid aglycones and glycosides fell into a characteristic RMD range (200–400 ppm) [73], and the maximum abundance peak was often that of the aglycone in-source fragment. Thus, the flavonoid glycosides and flavonoid aglycones were differentiated by their discrete retention time ranges (350–450 and 600–800 s, respectively). The lipids and phospholipids were characterized by their high RMD (600–800 ppm), *m/z* (400–900 Da), and rt (750–1050 s), although some smaller and more polar phospholipids were observed at earlier retention times. Annotation was partially guided by Gaussian model clusters to help select the class of a molecule based on similar molecules in the cluster. In addition to aiding in annotation, Gaussian model clusters can be used to generate composite variables based on the physicochemical properties of phytochemicals.

Putative assignments to phytochemical classes were confirmed by annotating individual peaks based on high-resolution mass matches to the METLIN mass spectrometry databases [74]. High-resolution mass searches were conducted within a 30-ppm mass window for proton and sodium adducts, the most common adducts observed on our LC-TOF system. The pseudospectra were checked for the presence of ammonium adducts, which were used in database searches if they were the most abundant peak. The phytochemical classes were defined as: peptide-like compounds, flavonoids and flavonoid glycosides, lipids, phospholipids, alkaloids, amino acids, and a small cluster of unknown compounds. Especially in the case of flavonoids, many isomeric and isobaric matches were found for a single peak *m/z*. A molecule was presumed to belong to the flavonoid class when the lowest ppm matching compounds were similar to known *Ceanothus* flavonoids, which are predominantly quercetin and kaempferol analogs, in accord with our observations [44]. The peptides were presumed to be database matches when the database hits were predominantly peptidic, whereas if many small molecule and peptidic compounds had mass matches, the predominant small molecule class was selected if it fell into a cluster with other compounds belonging to the same phytochemical class. Compounds identified as flavonoids, flavonoid glycosides, and peptides were normalized to naringenin, naringin, and cholecystokinin fragment 30–33 amide internal standards, respectively (all other compounds were not normalized) before normalizing to dry plant mass extracted (g). 

### 4.6. WGCNA Correlation Networks and Model Construction

All LC-TOF data processing and statistical analyses were conducted in the R statistical platform [75]. Groups of covarying phytochemicals were identified as modules resulting from correlation networks produced using the WGCNA package in R [30]. Unsigned correlations (include both positive and negative correlations) were raised to the power of eight based on the highest scale-free topology index value, the merge cut height was set to 0.25, and the minimum module size was set to five. Data were mean centered prior to WGCNA. The resulting network was visualized in Gephi [76] using ForceAtlas2 layout. 

All other analyses were carried out using peak abundance data normalized to appropriate internal standards and dry plant mass (*vide supra*). Measures of phytochemical and herbivore diversity, dispersion, and composition were generated in the R package vegan [77]. The phytochemical and herbivore diversity were calculated as Shannon entropy (*diversity* function). Herbivore diversity and abundance were calculated for all morphotyped Lepidopteran individuals at the plot level, aggregated over the three collection times. The phytochemical diversity was calculated at the individual level within each collection time before aggregating at each spatiotemporal level indicated. The phytochemical and herbivore dispersion were calculated (*betadisper* function) and differences in phytochemical and herbivore composition were calculated using Permutational Multivariate Analysis of Variance Using Distance Matrices (PERMANOVA) via the *adonis* function. For ADONIS and dispersion analyses, an interindividual phytochemical distance matrix was generated as Manhattan distance (*dist* function in core R), and an inter-plot herbivore dissimilarity matrix was generated as Jaccard similarity (*vegdist* in vegan). ADONIS analyses were not performed when there was a significant difference in dispersion to accommodate assumptions of equal variance. In this study, the dispersion of the herbivore community represents a measure of species turnover, or variation in community composition, between plots at the subsite and site levels. Dispersion of phytochemistry represents a measure of variation in the *C. velutinus* metabolome between individuals at different collection times at the month-within-plot, plot, subsite, and site level; dispersion at the plot level incorporates interindividual and temporal variation, and at the subsite and site level incorporates spatiotemporal variation as well.

The peak bins were partitioned into data subsets by module and dispersion analyses were conducted for each module set. Parasitoid success was calculated as the number of parasitoid-yielding lepidoptera that survived rearing for each plot. Leaf, herbivore, and parasitoid abundances were natural log transformed to meet assumptions of normality. Structural equation models were generated using the *sem* function in the R package lavaan [78]. Model fit was assessed based on the chi-square goodness of fit, wherein the *p* value represents the percent certainty that the model represents the actual relationships underlying the data (e.g., *p* = 0.90 means there is a 90% chance that the model is correct). Model fit was also assessed based on the Aikake Information Criterion (AIC), which penalizes models for higher numbers of variables and interactions. All other linear models, correlations, Student’s T-tests, F-tests of equal variance, and ANOVA were conducted in R. Correlation coefficients are reported as Pearson’s *r*. 

## Data Availability

Data is available in the Dryad Digital Repository: https://doi.org/10.5061/dryad.gqnk98sn7 (accessed on 31 March 2021).

## Supplementary Materials

The following are available online at https://www.mdpi.com/article/10.3390/metabo11060361/s1, Table S1. Ceanothus velutinus composition of plots at Dog Valley and Mt. Rose sites, Figure S1: Subsite-level C. velutinus abundance, Figure S2: Gaussian cluster model of partitioned odd and even mass features based on the physicochemical properties of 176 compounds found in C. velutinus, Figure S3: Bar plots showing the proportion of each compound class after cluster-guided annotation represented by Gaussian Model Clusters, Table S2: Mean and standard deviation of physicochemical properties of C. velutinus compounds within Gaussian model clusters, Figure S4: Herbivore dispersion and composition, phytochemical dispersion, and phytochemical diversity at the Dog Valley and Mt. Rose sites, Figure S5: Boxplots of chemical diversity at multiple levels, Figure S6: Boxplots of chemical dispersion at two different levels, Figure S7: Correlations between herbivore dispersion and ecological variables, Figure S8: Chemical dispersion of phytochemicals partitioned into modules across elevation.
