# Supplementary material for: Opposing Effects of Ceanothus velutinus Phytochemistry on Herbivore Communities at Multiple Scales
Source: Metabolites. 2021 Jun 7;11(6):361. doi: 10.3390/metabo11060361 (PMC8227664; doi:10.3390/metabo11060361)
Supplement: Supplementary file 1 [file metabolites-11-00361-s001.zip › metabolites-1226636-supplementary.pdf]

Supplemental Information

Table S1. *Ceanothus velutinus* composition of plots at Dog Valley and Mt. Rose sites.

Site	Subsite	Plot	Elevation	Leaf Abundance	Mean Leaves Per Plant[†]	Plant Abundance
Dog Valley	1	192	1632	10000	10000	1
	1	194	1656	10000	10000	1
	1	187	1659	13600	6800±6200	2
	2	193	1898	6215	390±90	16
	2	189	1939	10500	660±128	16
	2	186	1947	18290	590±90	31
	3	191	2182	7800	2600±945	3
	3	190	2185	11900	2980±2350	4
	3	188	2236	15000	15000	1
Mt. Rose	1	195	1824	14000	1750±350	8
	1	198	1837	8500	1700±780	5
	1	196	1839	3600	1800±600	2
	2	201	2162	1500	750±450	2
	2	203	2183	5150	1030±500	5
	2	202	2188	10500	2100±1090	5
	3	200	2525	2500	2500	1
	3	199	2541	45000	45000	1
	3	197	2550	9000	9000	1

[†] ±S.E.M.

Table S2. Mean and standard deviation of physicochemical properties of *C. velutinus* compounds within Gaussian model clusters.

Odd-mass compounds

	rt (sec)		*m/z* (Da)		RMD (ppm)			
Cluster	mean	sdev	mean	sdev	mean	sdev	# peaks	Predominant class
1	282	155	257	127	478.111	157.358	18	Peptide
2	725	126	272	105	341.000	102.720	13	Flavonoid aglycone
3	413	73	316	24	228.188	51.432	16	Flavonoid glycoside
4	938	104	553	53	815.455	95.225	22	Lipid
5	419	62	497	38	223.667	52.381	15	Flavonoid glycoside
6	390	74	582	104	326.636	70.305	11	Flavonoid glycoside
7	896	64	618	20	456.000	39.266	14	Peptide
8	858	319	885	103	486.182	184.383	11	Phospholipids
9	937	46	797	49	648.800	65.698	5	Phospholipids

Even-mass compounds

	rt (sec)		*m/z* (Da)		RMD (ppm)			
Cluster	mean	sdev	mean	sdev	mean	sdev	# peaks	Predominant class
1	510	290	439	161	635.667	155.669	24	Peptide
2	1062	46	572	70	863.500	48.483	18	Lipid
3	367	64	550	52	287.000	106.737	6	Peptide
4	1088	41	839	126	296.500	9.958	10	Unknown
5	996	47	784	25	728.250	34.773	20	Phospholipids
7	1086	43	296	11	864.922	103.396	9	Unknown

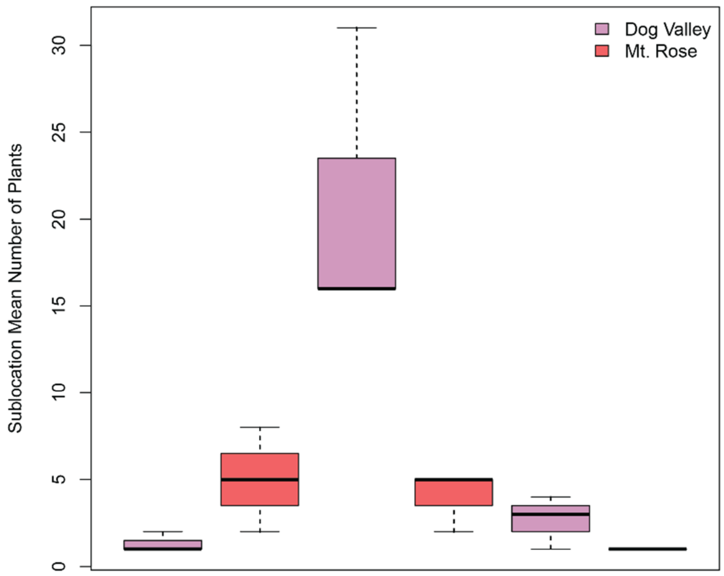

(a)

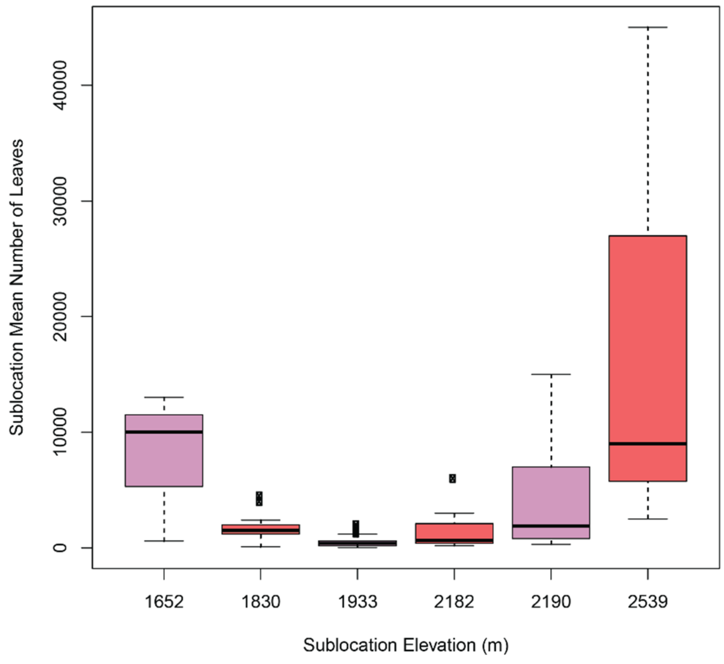

(b)

Figure S1. Subsite-level *C. velutinus* abundance. (a) Boxplot of the number of *C. velutinus* individuals by subsite across an elevational gradient. (b) Number of leaves at the subsite level across an elevational gradient. In Dog Valley, more established plants (more leaves) are found at lower elevations, with higher numbers of younger plants at higher elevations. In Mount Rose, more established plants are at higher elevations with younger populations at lower elevations.

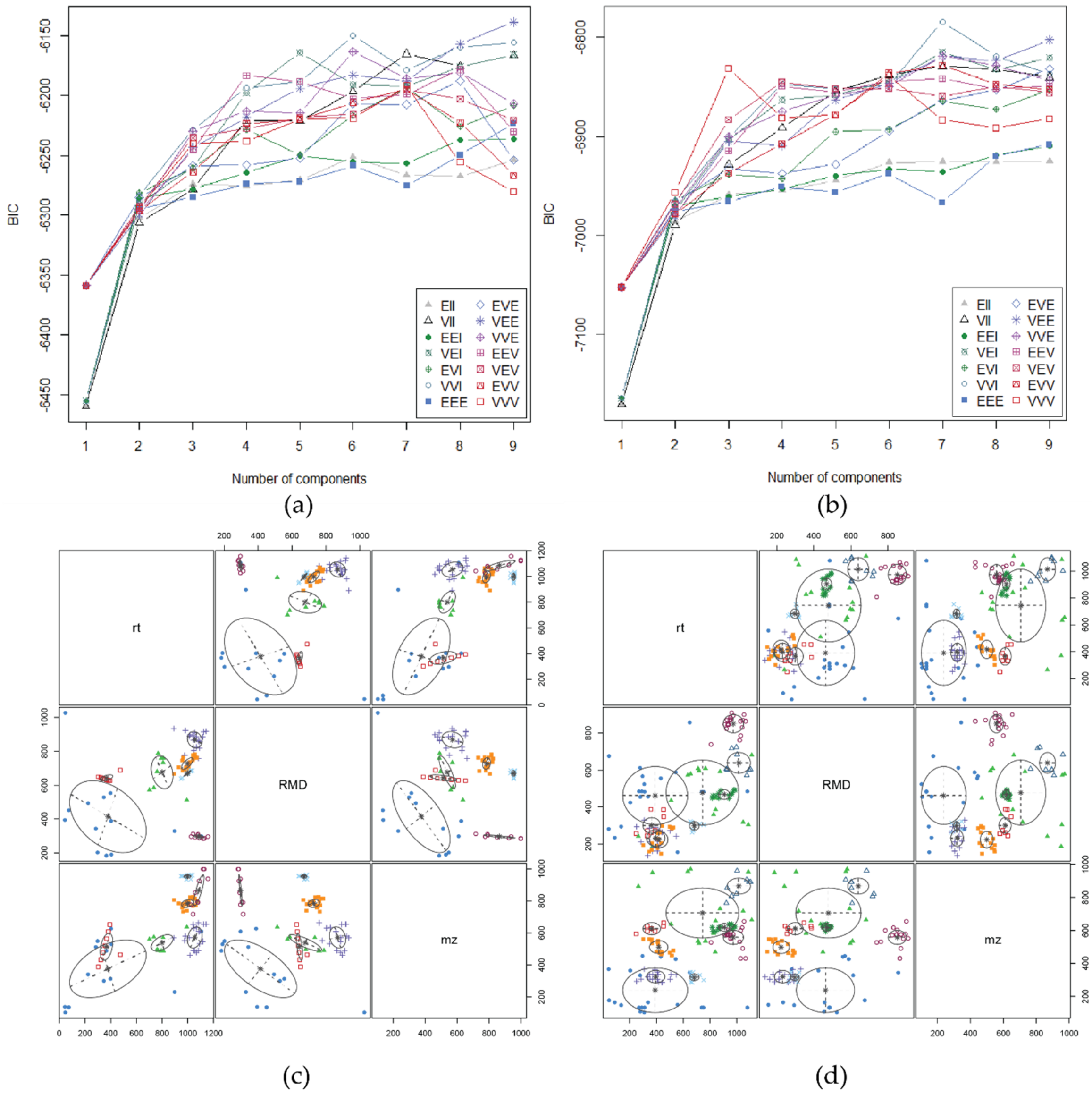

Figure S2. Gaussian cluster model of partitioned odd and even mass features based on the physicochemical properties of 176 compounds found in *C. velutinus*. **(a)** Model comparisons for odd *m*/*z* phytochemicals, model VEE and nine clusters were selected based on the highest Bayesian Information Criterion (BIC) . **(b)** Model comparisons for even *m*/*z* phytochemicals, model VVI with seven clusters were selected based on the highest BIC value. Models listed in the legend (e.g. VEE) represent different combinations of the geometric characteristics (distribution, volume, shape, and orientation) of clusters models. **(c)** Gaussian model cluster groups based on retention time (rt), relative mass defect (RMD) and mass-to-charge ratio (mz) of odd *m*/*z* phytochemicals. **(d)** Gaussian model cluster groups based on retention time (rt), relative mass defect (RMD) and mass-to-charge ratio (mz) of even *m*/*z* phytochemicals.

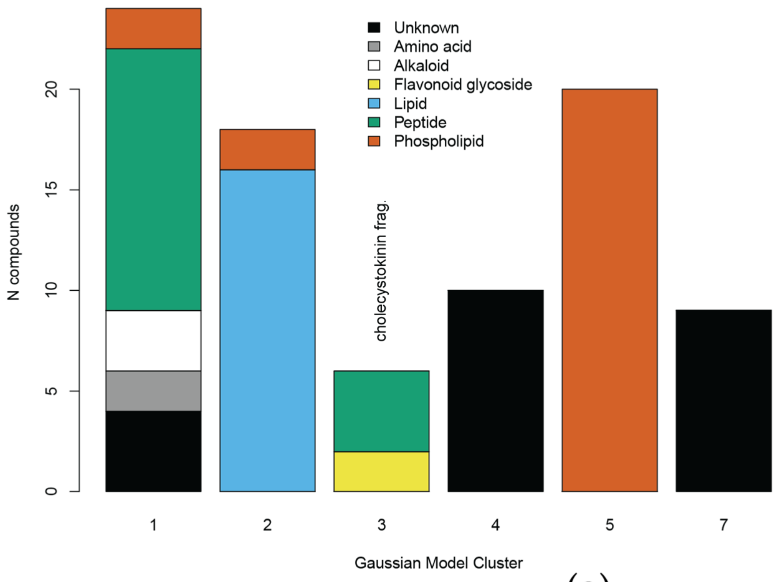

(a)

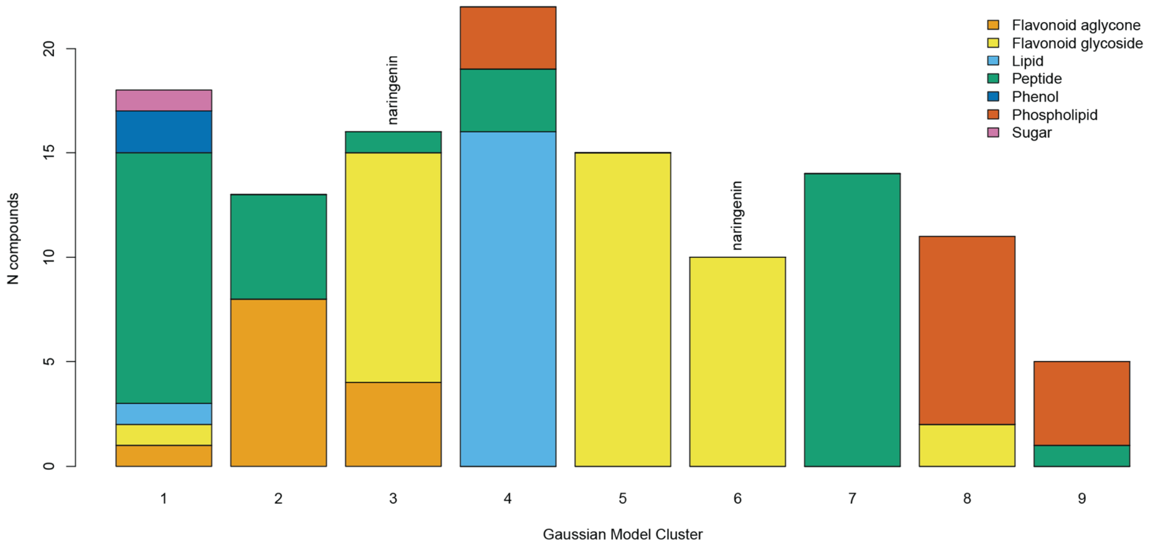

(b)

Figure S3. Bar plots showing the proportion of each compound class after cluster-guided annotation represented by Gaussian Model Clusters. **(a)** even *m*/*z* clusters representing presumably nitrogenous compounds. **(b)** odd *m*/*z* clusters representing presumably non-nitrogenous compounds

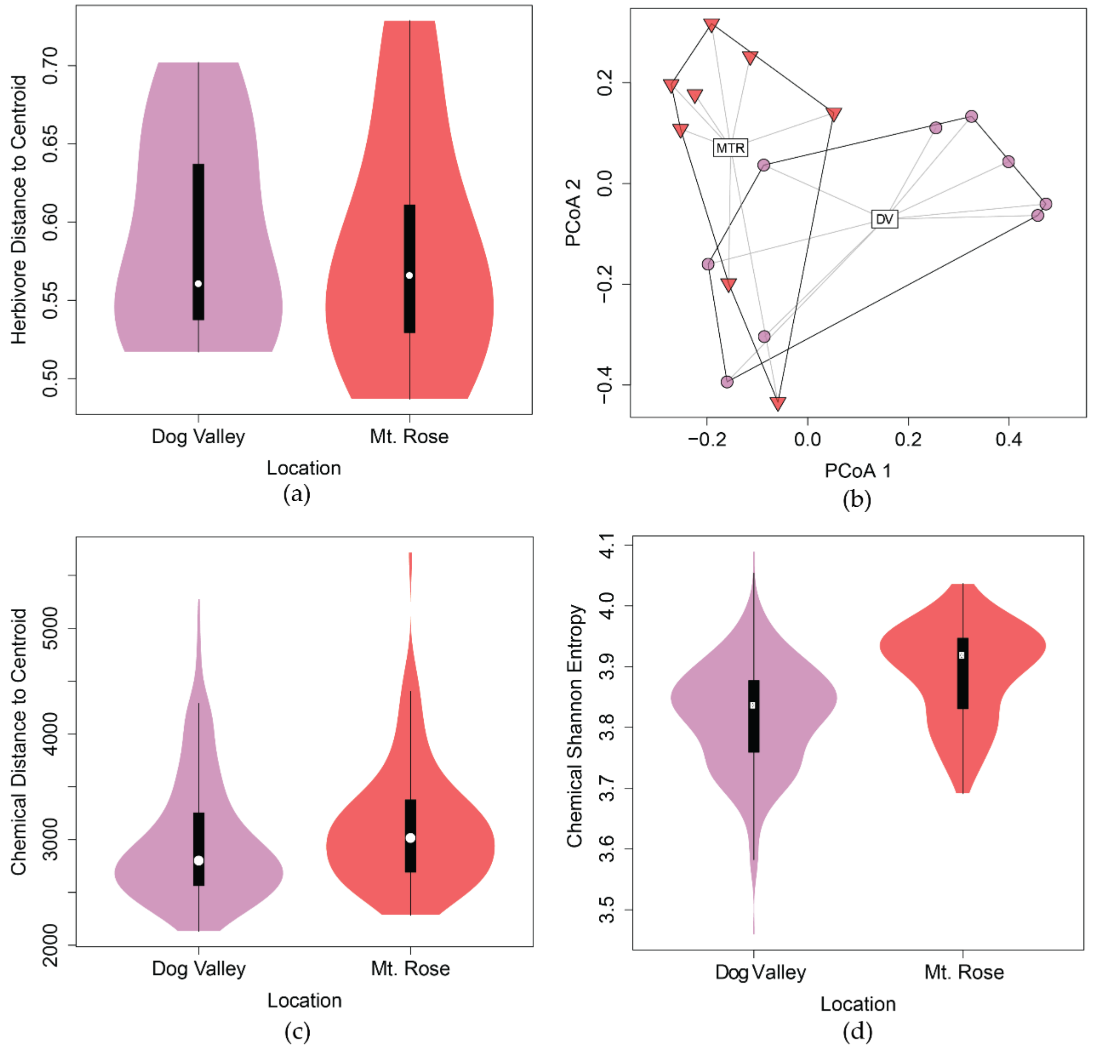

Figure S4. Herbivore dispersion **(a)** and composition **(b)**, phytochemical dispersion **(c)** and phytochemical diversity **(d)** at Dog Valley and Mt. Rose sites. Neither site differed significantly by herbivore or phytochemical dispersion ($ANOVA_{1,283}$ $p > 0.05$), but herbivore composition differed significantly ($ADONIS_{1,283}$ $p < 0.01$), as did mean phytochemical diversity (Shannon entropy, $ANOVA_{1,283}$ $p > 0.001$). MTR: Mt. Rose site; DV: Dog Valley. The principal coordinate analysis (PCoA, Jaccard) plot in **b** shows how herbivore composition differs between the two sites, but not herbivore variance.

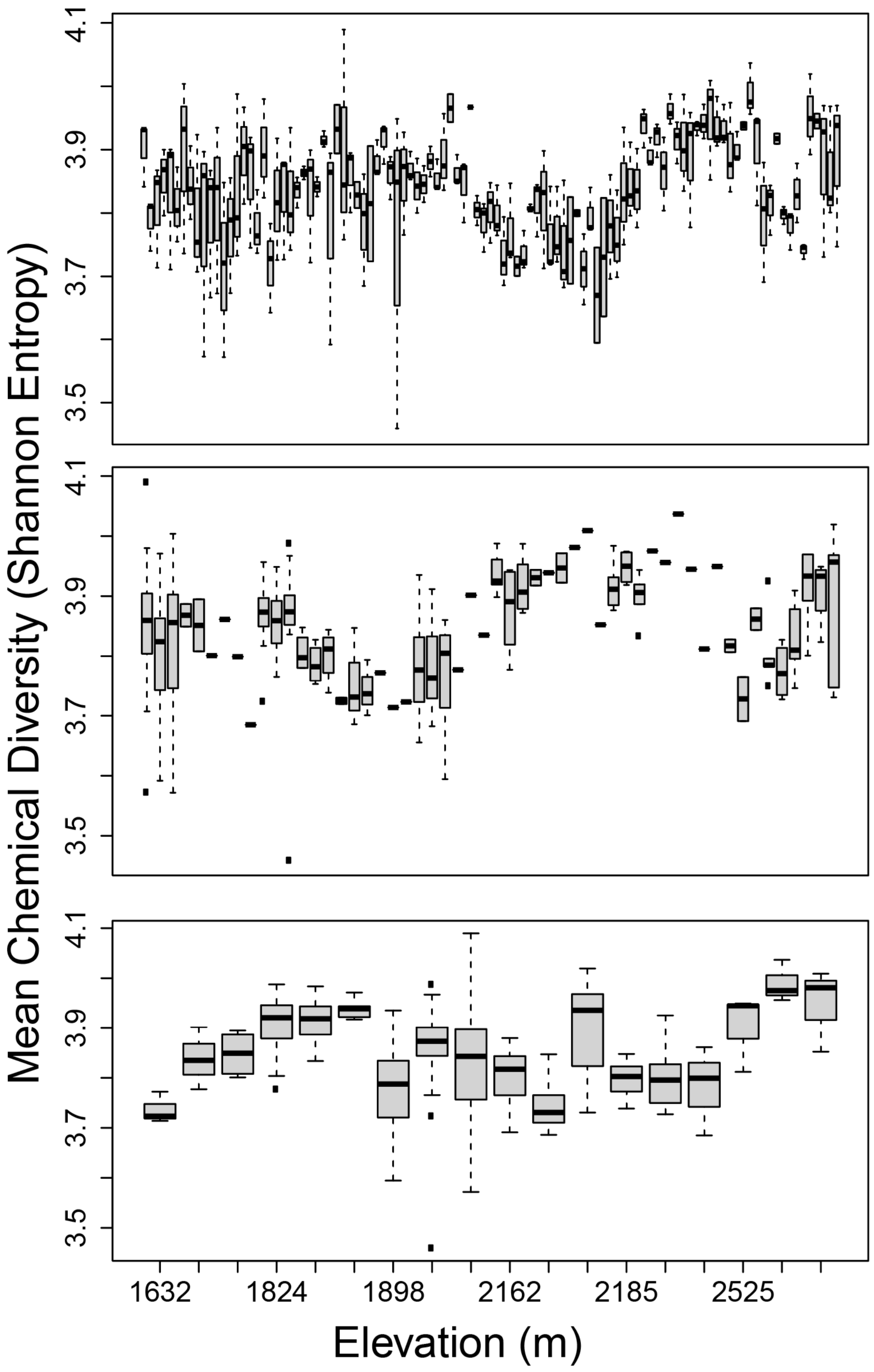

Figure S5. Boxplots of chemical diversity at multiple levels. Mean chemical diversity varied significantly at the **(a)** individual across month ($ANOVA_{104,196}$; $p < 0.001$), **(b)** collection month within-plot across individual ($ANOVA_{53,247}$; $p < 0.001$), and **(c)** plot ($ANOVA_{5,295}$; $p < 0.001$) levels. All levels of phytochemical diversity showed a sinusoidal relationship with elevation.

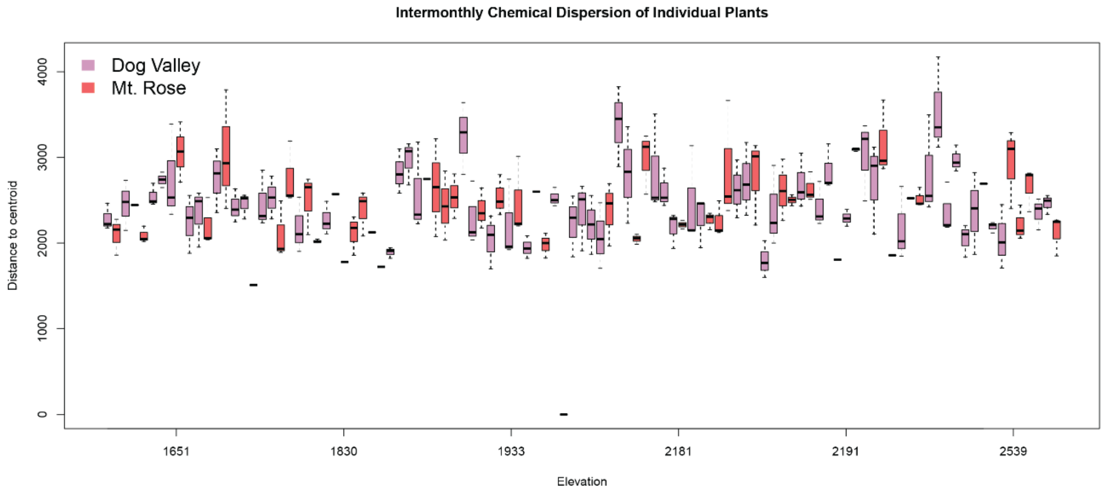

(a)

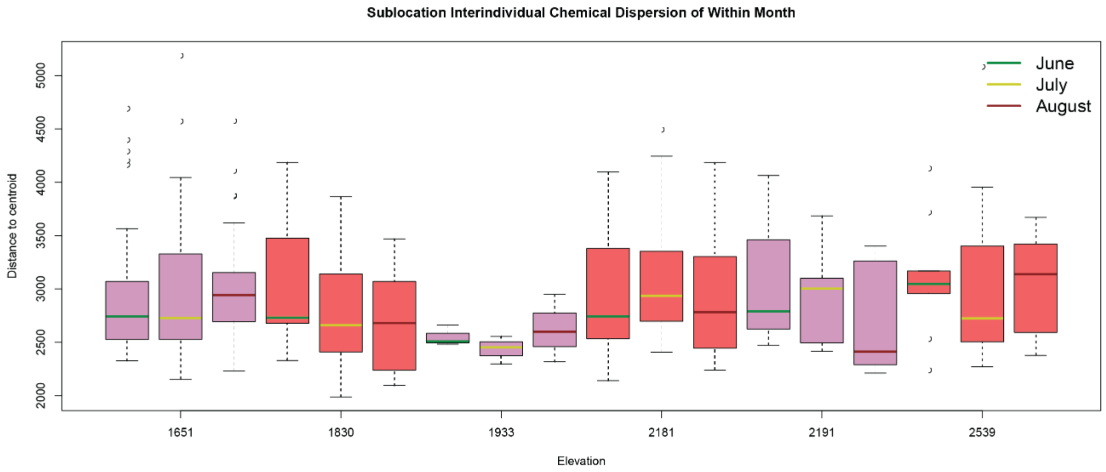

(b)

Figure S6. Boxplots of chemical dispersion at two different levels. **(a)** Inter-monthly chemical dispersion of individual plants across an elevational gradient ($ANOVA_{104,196}$ $p < 0.001$). **(b)** Subsite-level interindividual dispersion by collection month across an elevational gradient ($ANOVA_{17,283}$ $p < 0.05$).

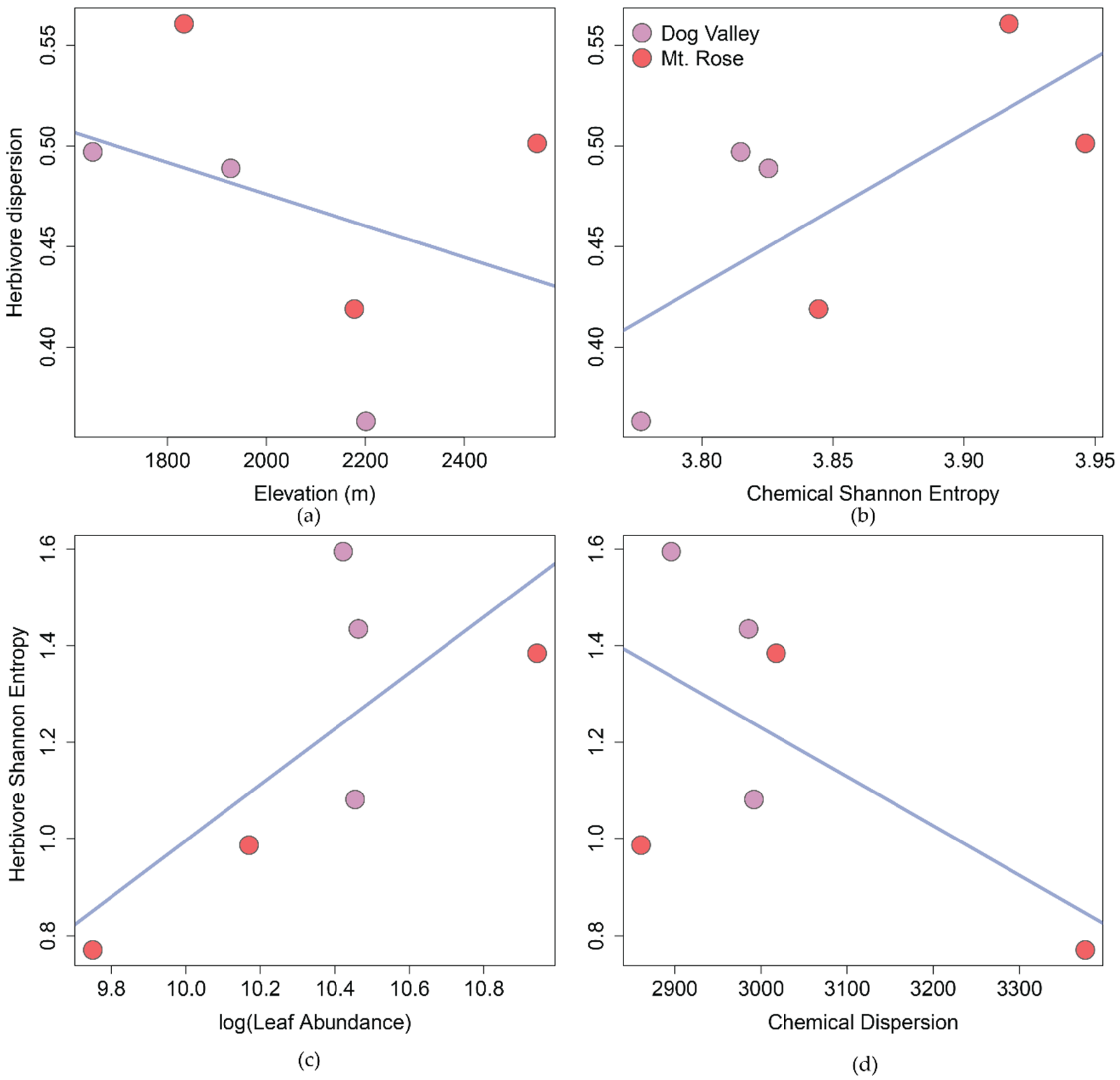

Figure S7. Correlations between herbivore dispersion and ecological variables. Herbivore dispersion (mean distance from centroid) is negatively ($r[6] = -0.60$, $p < 0.05$) correlated with **(a)** elevation and positively correlated ($r[6] = 0.73$, $p < 0.01$) with **(b)** phytochemical diversity (Shannon Entropy). Herbivore diversity (Shannon Entropy) is positively correlated ($r[6] = 0.69$, , $p = 0.10$) with **(c)** natural log-transformed leaf abundance and negatively correlated ($r[6] = -0.66$, $p = 0.21$) with **(d)** phytochemical dispersion (mean distance from centroid).

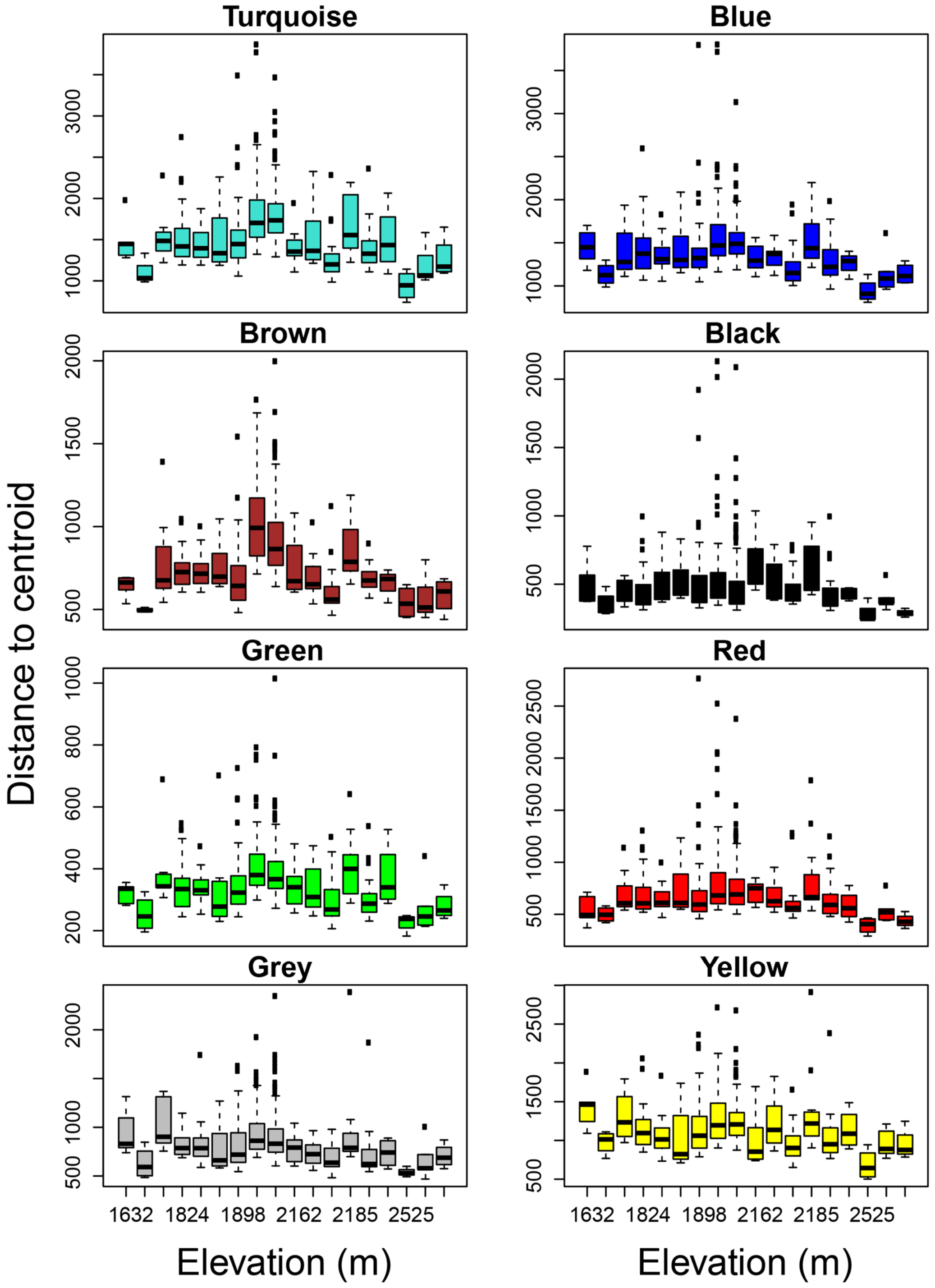

Figure S8. Chemical dispersion of phytochemicals partitioned into modules generated from WGCNA across elevation differs between plots. The highest F-statistic values were found for the brown, green and turquoise modules ($ANOVA_{17,431}$; $F_{brown} = 13.9$, $F_{green} = 4.7$, $F_{turquoise} = 4.3$), while the remaining modules having significant dispersion ($ANOVA_{17,431}$; $p < 0.01$) had F-statistic values ranging from 2.3-3.5. The black module that did not have significant between-plot dispersion ($ANOVA_{17,431}$; $p > 0.1$).
